# CaCDPK15 positively regulates pepper responses to *Ralstonia solanacearum* inoculation and forms a positive-feedback loop with CaWRKY40 to amplify defense signaling

**DOI:** 10.1038/srep22439

**Published:** 2016-03-01

**Authors:** Lei Shen, Sheng Yang, Tong Yang, Jiaqi Liang, Wei Cheng, Jiayu Wen, Yanyan Liu, Jiazhi Li, Lanping Shi, Qian Tang, Wei Shi, Jiong Hu, Cailing Liu, Yangwen Zhang, Shaoliang Mou, Zhiqin Liu, Hanyang Cai, Li He, Deyi Guan, Yang Wu, Shuilin He

**Affiliations:** 1 National Education Ministry, Key Laboratory of Plant Genetic Improvement and Comprehensive Utilization, Fujian Agriculture and Forestry University, Fuzhou, Fujian 350002, PR China; 2 College of Crop Science, Fujian Agriculture and Forestry University, Fuzhou, Fujian 350002, PR China; 3 College of Life Science, Fujian Agriculture and Forestry University, Fuzhou, Fujian 350002, PR China; 4 College of Life Science, Jinggang Shan University, Ji’an, Jiangxi 343000, PR China

## Abstract

CaWRKY40 is a positive regulator of pepper (*Capsicum annum*) response to *Ralstonia solanacearum* inoculation (RSI), but the underlying mechanism remains largely unknown. Here, we functionally characterize *CaCDPK15* in the defense signaling mediated by CaWRKY40. Pathogen-responsive TGA, W, and ERE boxes were identified in the *CaCDPK15* promoter (p*CaCDPK15*), and p*CaCDPK15*-driven GUS expression was significantly enhanced in response to RSI and exogenously applied salicylic acid, methyl jasmonate, abscisic acid, and ethephon. Virus-induced gene silencing (VIGS) of *CaCDPK15* significantly increased the susceptibility of pepper to RSI and downregulated the immunity-associated markers *CaNPR1*, *CaPR1*, and *CaDEF1*. By contrast, transient *CaCDPK15* overexpression significantly activated hypersensitive response associated cell death, upregulated the immunity-associated marker genes, upregulated *CaWRKY40* expression, and enriched CaWRKY40 at the promoters of its targets genes. Although CaCDPK15 failed to interact with CaWRKY40, the direct binding of CaWRKY40 to p*CaCDPK15* was detected by chromatin immunoprecipitation, which was significantly potentiated by RSI in pepper plants. These combined results suggest that RSI in pepper induces *CaCDPK15* and indirectly activates downstream *CaWRKY40*, which in turn potentiates *CaCDPK15* expression. This positive-feedback loop would amplify defense signaling against RSI and efficiently activate strong plant immunity.

Exposure to biotic or abiotic stresses triggers transcription reprogramming of defense-associated gene expression and subsequent biochemical and physiological responses, which leads to plant adaptation^1^,^2^. Accumulating evidence indicates that defense-associated transcription reprogramming is tightly regulated by complex signaling networks, which begin with the perception and recognition of stress signals at the plasma membrane, and cumulate with the transcriptional modification of defense-associated gene expression in the nucleus^2^,^3^. Genetic modification of some signaling cascades within this transcriptional network can produce significant phenotypic effects^4^,^5^. Unraveling this network is one of the most important tasks in plant biology. However, the hierarchical organization, coordination, and fine-tuning of these signaling networks remain to be elucidated.

Calcium (Ca^2+^) is a ubiquitous second messenger involved in plant responses to abiotic and biotic stresses^6^. Stress triggers Ca^2+^ flux across the plasma membrane, leading to rapid increases in the cytoplasmic Ca^2+^ concentration, with stress-dependent variations in frequency, amplitude, and duration^7^,^8^. These changes in Ca^2+^ concentration are sensed and decoded by different Ca^2+^ sensors and/or Ca^2+^ -binding proteins, including calmodulins (CaMs), calmodulin-like proteins (CaMLs), calcineurin B-like proteins (CBLs), and calcium-dependent protein kinases (CDPKs), which subsequently activate different downstream defense responses^5^,^6^,^9^. CDPKs are characterized by an N-variable domain, a protein kinase domain, an autoinhibitory domain, and a CaM-like domain^10^. These unique features enable CDPKs to function as Ca^2+^ sensors and effectors, thereby allowing individual CDPK proteins to relay specific Ca^2+^ signatures to downstream components via CDPK-dependent protein phosphorylation. CDPKs are encoded by a large gene family, including 34 *CDPK* genes in *Arabidopsis*^10^, 31 in rice^11^, 40 in maize^12^, 20 in wheat^13^, 30 in *Populus*^14^, 41 in cotton^15^, and 35 in pepper. Many CDPK family members in *Arabidopsis* have been reported or predicted to be membrane-associated proteins^16^. A subset of *CDPK* genes exhibit inducible expression patterns; have pivotal roles in plant responses to abiotic stresses such as drought^17^,^18^,^19^, cold^17^,^18^,^19^, salinity^18^,^20^, heat shock^21^, dehydration^19^, and arsenic stress^22^; function in plant responses to biotic stresses such as pathogen infection^23^ and herbivore attack^24^; and have key roles in reactive oxygen species (ROS) signaling^23^. Currently, the functional identification of CDPKs has focused on a few family members or a few plant species. The roles and functional mechanisms of the majority of CDPK family members in diverse plant species remain to be elucidated.

WRKY proteins constitute one of the largest transcription factor (TF) families, characterized by their highly conserved WRKY domains and indiscriminate binding to their cogent W-box^25^. WRKY members in different plant species have been implicated in responses to abiotic and biotic stresses, including drought^26^,^27^, salt^26^, cold^28^, heat^27^, pathogen^28^ and virus^29^ infection, and herbivore attack^30^. The mechanism underlying WRKY TF-mediated defense responses appears to be very complex. A subset of *WRKY* genes are frequently induced by single stresses, and *WRKY* promoters are frequently enriched for W-boxes, which appear to be constitutively occupied by WRKY TFs^31^. This indicates that multiple WRKY TFs and transcriptional networks are involved in plant stress responses^32^. Alternatively, one WRKY TF may respond to multiple stresses, which would function as a crucial node in the crosstalk between responses to biotic and abiotic stresses, or responses to different stresses. A WRKY TF might have specific patterns of expression and function in specific contexts, although the underlying mechanisms are unknown. The binding of WRKY TFs to their specific W-box targets is affected by the adjacent DNA sequences outside of the TTGACY-core motif^33^ and the W-box consensus is degenerate; the majority of analyzed WRKY proteins recognize the TTGACC/T W-box sequences. Therefore, other components are believed to be required by WRKY TFs to mediate stimulus-specific responses^33^,^34^. WRKY TFs have protein-protein interactions with MAPKs, CDPKs, 14-3-3 proteins, VQ domain-containing proteins, and NAC (NAM/ATAF/CUC) TFs, which modify WRKY TF activities and modulate important biological processes^34^,^35^. However, the molecular linkages between nuclear-localized WRKY TFs and their interactions with upstream cytoplasmic signaling factors remain to be elucidated.

Pepper (*Capsicum annuum*) is one of the most important vegetables worldwide. Pepper is a typical *Solanaceae* and is susceptible to various soil-borne diseases such as *Ralstonia solanacearum* inoculation (RSI) and *Phytophthora capsici*, which is aggravated by high temperature and high humidity. These diseases cause heavy losses in crop yield and quality. Pepper germplasm evolved under these stresses, and may have developed unique mechanisms for disease tolerance and/or resistance, and tolerance to high temperature and high humidity. Our previous study reported that CaWRKY40 functioned as positive regulator in pepper’s response to *Pseudomonas solanacearum* attack and heat stress under high humidity^36^, and *CaWRKY40* was transcriptionally modulated by CaWRKY6, another member in the WRKY TF family in pepper^37^. In the present study, the pepper CDPK protein designated as CaCDPK15 was found to regulate pepper response to *Pseudomonas solanacearum* attack by indirectly activating *CaWRKY40* expression, and CaWRKY40 in turn activated transcriptional expression of *CaCDPK15* by binding to the *CaCDPK15* promoter, thereby establishing a positive-feedback loop.

## Results

### p*CaCDPK15-*driven GUS expression was upregulated by RSI and exogenously applied signaling mediators in pepper plants

**Figure 1:**
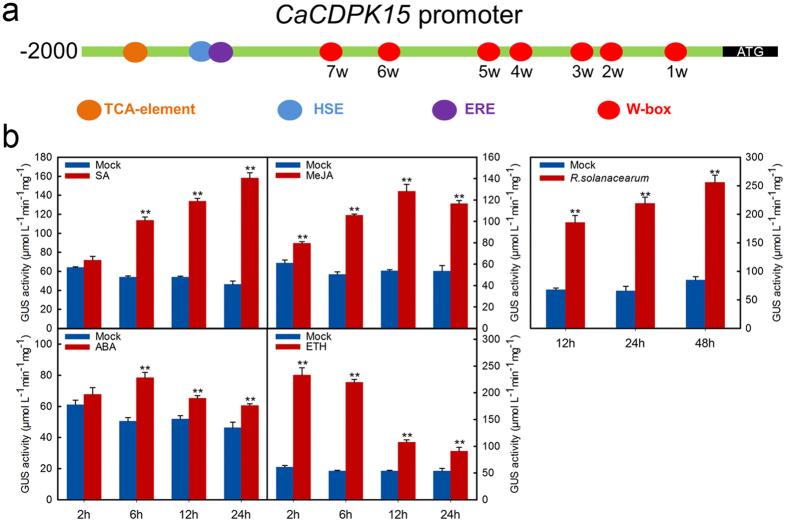
The expression of p*CaCDPK15*-driven GUS was induced by hormones and RSI. (**a**) The main *cis*-elements including one TCA-element, one HSE, one ERE and seven W-boxes in p*CaCDPK15*. (**b**) The p*CaCDPK15* driven GUS expression was promoted by exogenous application of SA, MeJA, ETH and ABA and RSI. The leaves of pepper plants at eighty-leaf stage ware infiltrated with GV3101 cells (OD_600_ = 0.6) containing p*CabZIP63:GUS*, and 24 hours later the plants were treated with 1 mm SA, 100 μm MeJA, 100 μm ETH, 100 μm ABA, or inoculated with the *R. solanacearum* (OD_600_ = 0.6). The leaves were harvested at different time points for GUS activity assay by microplate reader using pepper leaves treated with ddH_2_O as mock. Data are the means ± SD from at least three independent experiments. Asterisks indicate statistically significant differences compared with Mock (treated with ddH_2_O). (*t*-test, **P < 0.01).

Our previous study performed a genome-wide identification of pepper CDPK members. A total of 35 *CDPK* genes were identified in the genome of the pepper variety CM334 using genome sequence data^38^ (DOI:10.3389/fpls.2015.00737). The *CaCDPK15* gene contained an N-variable domain, a protein kinase domain, an autoinhibitory domain, and a CaM-like domain, which are unique features of CDPK proteins^10^ (Supplementary Fig. S1). *CaCDPK15* exhibited inducible transcriptional expression in response to RSI (DOI:10.3389/fpls.2015.00737), suggesting a possible role in pepper immunity to *R. solanacearum*. In the present study, the *CaCDPK15* promoter (p*CaCDPK15*) was identified, along with the *cis*-elements within the promoter (1 TCA element, 1 HSE, 1 ERE, and 7 W-boxes) (Fig. 1a). A p*CaCDPK15*-driven GUS reporter was expressed in pepper leaves by agroinfiltration, and GUS expression in pepper leaves was measured in response to RSI and exogenous application of salicylic acid (SA), methyl jasmonate (MeJA), abscisic acid (ABA), and ethephon (ETH). The results showed that GUS expression was upregulated by RSI, SA, MeJA, ABA, and ETH, with different temporal expression patterns (Fig. 1b).

### CaCDPK15 is localized to the plasma membrane and nucleus

**Figure 2:**
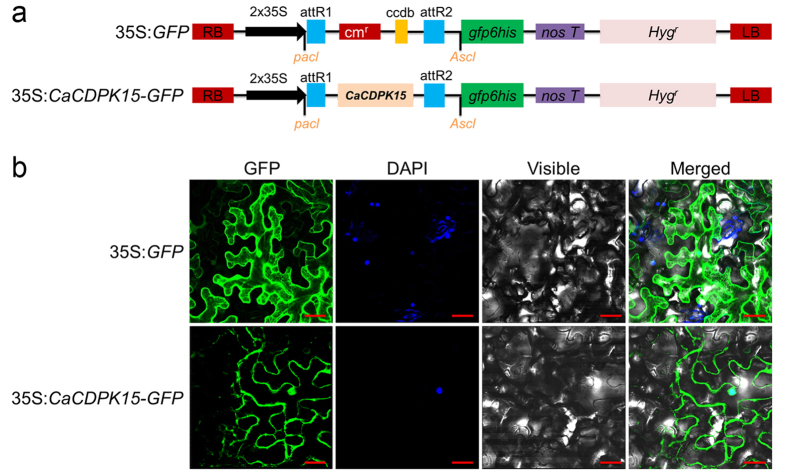
Subcellular location of *CaCDPK15*. (**a**) Schematic representations of the vector constructs of 35S:*GFP* and 35S:*CaCDPK15-GFP*. (**b**) GFP fusion protein of CaCDPK15 was localized in the cytomembrane and nucleus of *N. benthamiana* cells. The plant nuclei were stained with DAPI. Images were taken by using Leica confocal microscopy at 48 hours post agroinfiltration (GFP fluorescence, green; DAPI fluorescence, blue; visible, visible light image; merged, merged images of above three images). Bars = 200 μm.

The subcellular localization of a protein can determine or influence its function. To determine the subcellular localization of CaCDPK15, we generated a CaCDPK15-GFP fusion construct driven by the constitutive CaMV35S promoter, and expressed the construct in *Nicotiana benthamiana* leaves by agroinfiltration. The subcellular locations of CaCDPK15-GFP and GFP control were visualized with laser scanning confocal microscopy. The results revealed that CaCDPK15-GFP was localized in both the plasma membrane and the nucleus, whereas the GFP control was localized in multiple subcellular compartments including the cytoplasm and the nucleus (Fig. 2).

### Effect of *CaCDPK15* silencing on pepper resistance to RSI

**Figure 3:**
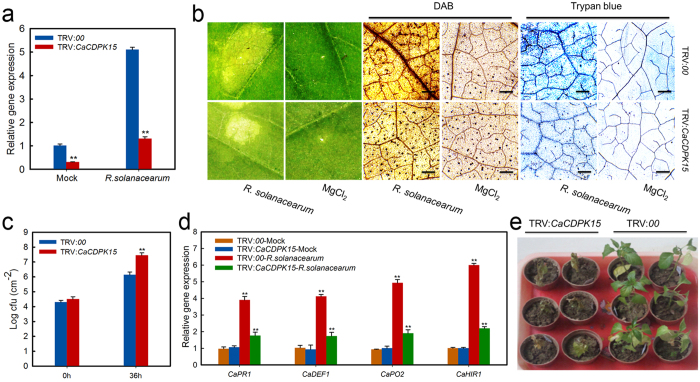
Enhanced susceptivity of *CaCDPK15*-silenced pepper plants to *R. solanacearum* inoculation. (**a**) Relative transcriptional expression of *CaCDPK15* in *CaCDPK15*-silenced pepper plants by real-time RT-PCR 24 h after *R. solanacearum* inoculation or the MgCl_2_ treatment (Mock). (**b**) Trypan blue staining and DAB staining in *R. solanacearum* inoculated *CaCDPK15*-silenced (TRV:*CaCDPK15*) and empty vector (TRV:*00*) pepper leaves at 2 days post inoculation. Bars = 50 μm. (**c**) Detection of *R. solanacearum* growth in *CaCDPK15*-silenced or the control pepper plants inoculated by *R. solanacearum* at 36 hours post inoculation. (**d**) Relative transcriptional expression of the defense marker genes by real-time RT-PCR in *CaCDPK15*-silenced pepper plants inoculated by *R. solanacearum* and the mock at 24 hpi. (**e**) Susceptivity analysis of *CaCDPK15*-silenced (TRV:*CaCDPK15*) and empty vector (TRV:*00*) pepper plants to *R. solanacearum* inoculation at 10 days post inoculation. *CaPR1*, pepper basic PR-1; *CaPO2*, peroxidase; *CaDEF1*, defensin; *CaHIR1*, hypersensitive induced reaction protein. Expression values are normalized by the expression levels of *CaACTIN* and *18s rRNA*. (**a**,**c**,**d**) Data are the means ± SD from at least three independent experiments. Asterisks indicate statistically significant differences compared with TRV:*00* and the treatment of MgCl_2_ (Mock). (*t*-test, **P < 0.01).

To test the role of *CaCDPK15* in pepper immunity, we evaluated CaCDPK15 loss-of-function in pepper seedlings by performing virus-induced gene silencing (VIGS). The vectors TRV1 (PYL192) and TRV2:*CaCDPK15* (PYL279) were separately transformed separately into *Agrobacterium tumefaciens* GV3101, and the two resulting GV3101 strains were mixed and co-infiltrated into pepper seedling leaves. The infiltrated seedlings were incubated at 16 °C for 56 h without light, and then were kept at 25 °C. Six independent experiments were performed, and we obtained approximately 100 plants of TRV:*00* and 100 plants of TRV:*CaCDPK15*. A TRV:*PDS* control construct was used in the same way to monitor gene silencing by the resulting photobleaching phenotype^36^. Six plants were randomly selected to check the gene silencing efficiency. In TRV:*CaCDPK15* pepper plants, *CaCDPK15* transcript levels were reduced to ~30% of those in TRV:*00* plants (Fig. 3a). The *R. solanacearum* strain FJC100301 was used to inoculate six individual TRV:*CaCDPK15* plants and six individual TRV:*00* empty vector control plants. We stained *R. solanacearum*-infected *CaCDPK15*-silenced and control leaves with DAB (indicator of H_2_O_2_ accumulation) and trypan blue (indicator of cell death or necrosis). Strongly polymerized DAB (dark brown) and hypersensitive response (HR)-mimicking cell death were detected in control leaves at 48 h post inoculation (hpi), whereas the intensities of DAB and trypan blue staining were distinctly reduced in *CaCDPK15*-silenced leaves (Fig. 3b). Our data also showed that *R. solanacearum* growth was significantly increased in *CaCDPK15*-silenced pepper plants, manifested by higher colony-forming units (cfu) compared with that in control plants (Fig. 3c). The expression of known pepper defense genes involved in the response to pathogen infection was analyzed by quantitative real-time PCR (qPCR) analysis. The results showed that transcript levels of the defense-related pepper genes *CaPR1*^39^, *CaDEF1*^40^, *CaPO2*^41^, and *CaHIR1*^42^ were lower in *CaCDPK15*-silenced leaves than in leaves of control pepper plants at 24 hpi (Fig. 3d). At 14 days post inoculation (dpi), we observed definite wilting symptoms on TRV:*CaCDPK15* pepper leaves, but the TRV:00 empty-vector control leaves exhibited only faint wilting symptoms (Fig. 3e).

### Transient *CaCDPK15* expression induces the hypersensitive response, cell death, and H_2_O_2_ accumulation in pepper leaves

**Figure 4:**
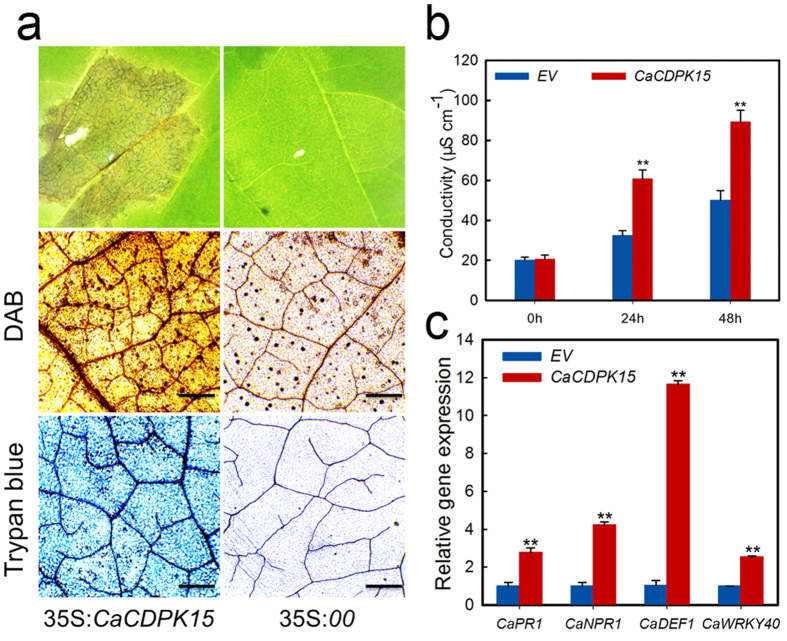
HR cell death and immunity was significantly triggered by transient overexpression of *CaCDPK15* in pepper leaves. (**a**) Cell death was found in pepper leaves at 4 days after infiltration with *Agrobacterium* GV3101 cells carrying 35S:*CaCDPK15*, GV3101 cells carrying an empty vector (35S:*00*) was used as a control. Trypan blue staining and DAB staining of pepper leaves transiently overexpressing 35S:*CaCDPK15* or 35S:*00* at 4 days post agro-infiltration. Bars = 50 μm. (**b**) Electrolyte leakages of leaf discs of pepper leaves infiltrated with GV3101 cells carrying 35S:*CaCDPK15* or 35S:*00* at 0, 24 or 48 hpi. **(c)** Relative expression of immunity associated genes and *CaWRKY40* in the pepper leaves transiently overexpressing 35S:*CaCDPK15* or empty vector at 24 hpi. *CaNPR1*, non-expresser of pathogenesis-related gene. Expression values are normalized by the expression levels of *CaACTIN* and *18s rRNA*. (**b**,**c**) Data are the means ± SD from at least three independent experiments. Asterisks indicate statistically significant differences compared with 35S:*00*. (*t*-test, **P < 0.01).

We attempted to generate transgenic *CaCDPK15*-overexpressing tobacco plants, but found that *CaCDPK15* overexpression was lethal in transgenic tobacco. Therefore, a transient overexpression system for *CaCDPK15* was generated by agroinfiltration of 35S:*CaCDPK15* or 35S:*00* (empty vector) into pepper leaves (Fig. 4a). HR-mediated cell death and H_2_O_2_ accumulation were assessed by staining with trypan blue to identify necrotic cells and DAB, respectively. The 35S:*CDPK15* construct distinctly induced a necrotic response in pepper leaves and H_2_O_2_ accumulation, whereas the empty-vector control did not induce a necrotic response and resulted in only weak DAB staining. We also performed an ion leakage test to analyze the severity of plasma membrane damage and thereby the severity of cell necrosis in cells transiently expressing *CaCDPK15*. Pepper leaves transiently overexpressing *CaCDPK15* exhibited more ion leakage at 24 and 48 h after agroinfiltration than that in leaves expressing the empty vector control (Fig. 4b). Real-time RT-PCR analysis of *CaCDPK15* transcripts in the transient expression system showed that transcripts were higher in leaves expressing 35 S:*CaCDPK15* than in empty-vector control leaves (Fig. 4c). We also examined changes in the expression of defense-related genes including SA-responsive *CaPR1* and *CaNPR1*^43^, JA-responsive *CaDEF1*, and *CaWRKY40* in the transient expression system. The results showed that the relative transcript levels of *CaPR1*, *CaNPR1*, *CaDEF1*, and *CaWRKY40* increased continuously during transient expression of *CaCDPK15*.

### The effect of transient overexpression of *CaCDPK15* on the binding of CaWRKY40 to its target genes

**Figure 5:**
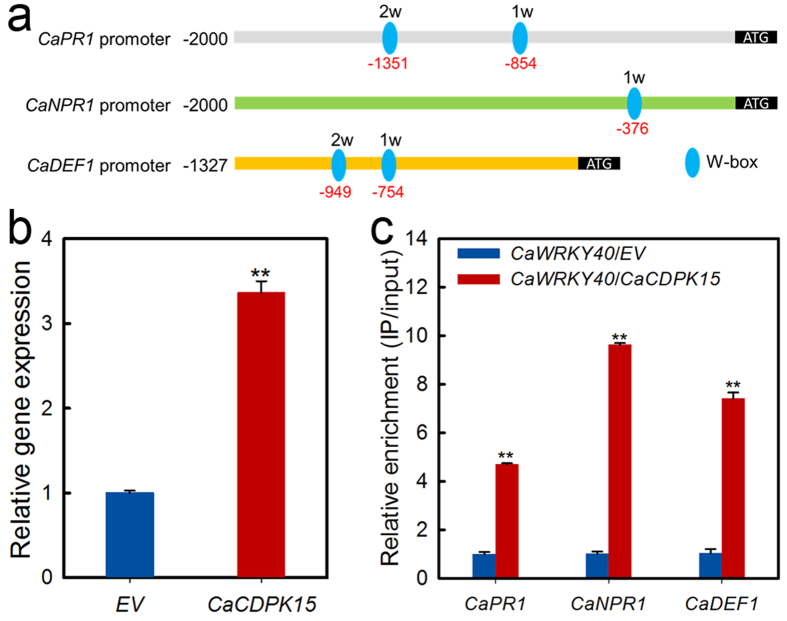
The transcriptional expression of *CaWRKY40* and the bindings of CaWRKY40 to the promoters of its target genes were enhanced by transient expression of *CaCDPK15*. (**a**) Schematic representation of the typical W-boxes in the promoters of the target genes of *CaWRKY40*. (**b**) The transcript level of *CaCDPK15* by transient overexpression itself in pepper leaves. GV3101 cells containing the construct of 35S:*CaCDPK15* was infiltrated into pepper leaves, which were harvest at 24 hpi to isolate the total RNA for transcriptional expressional assay of *CaCDPK15* by real-time RT-PCR. Expression values are normalized by the expression levels of *CaACTIN* and *18s rRNA.* (**c**) The bindings of CaWRKY40 to the promoters of its target genes were potentiated by transient expression of 35S:*CaCDPK15* in pepper plants. The GV3101 cells carrying the construct of 35S:*CaWRKY40-HA* and that containing 35S:*CaCDPK15-Flag* were mixed at a ratio of 1:1 and were co-infiltrated into pepper leaves, with GV3101 cells containing 35S:*00* as mock. The leaves were harvested at 48 hpi for chromatin preparation, the isolated chromatins were digested with micrococcal nuclease and the acquired DNA collections with 300–500 bp in length were used as templates for real-time RT-PCR to assay the bindings of CaWRKY40 to the promoters of its target genes, for each target gene of *CaWRKY40*, a specific primer pair flanking each typical W-box was designed and the one (primer pair based on 1 W in *CaPR1* promoter, 1 W in *CaNPR1* promoter and 2 W in *CaDEF1* promoter, respectively) that amplified product was used in the real-time RT-PCR analysis. Relative enrichment levels of samples of the CaWRKY40 transient overexpression were set to 1 after normalization by input. (**a,b**) Data are the means ± SD from at least three independent experiments. Asterisks indicate statistically significant differences compared with 35S:*00* (*EV*) and 35S:*CaWRKY40*/35S:*00* (*EV*). (*t*-test, **P < 0.01).

CaCDPK15 may modify CaWRKY40 transcriptional activity by altering its binding to the promoters of target genes. We tested this hypothesis by performing chromatin immunoprecipitation (ChIP) experiments. A specific primer pair was designed based on the flanking sequences of each typical W-box in the promoters of *CaPR1*, *CaNPR1*, and *CaDEF1* (Fig. 5a). For promoters with more than one W-box, the primer pairs were screened for product amplification and used in the real-time RT-PCR measurements of specific CaWRKY40 binding to the promoter. The results showed that CaWRKY40 binding to the promoters of *CaPR1*, *CaNPR1*, and *CaDEF1* was significantly enhanced by transient *CaCDPK15* overexpression (Fig. 5b,c).

### Detection of potential interactions between CaCDPK15 and CaWRKY40 by co-immunoprecipitation analysis

If CaCDPK15 acts as an upstream modifier of CaWRKY40 signaling, one possibility might be that CaWRKY40 is a target of CaCDPK15. We tested this hypothesis by performing co-immunoprecipitation (co-IP) analyses to evaluate possible interactions between the two proteins. These experiments employed a transient coexpression system in *N. benthamiana* leaves with the tagged constructs 35S:*CaCDPK15-HA* and 35S:*CaWRKY40-Flag*, and the positive control constructs 35S:*CaPIK1-Flag* and 35S:*CaSGT1-HA*^44^. The results showed that CaWRKY40 does not interact with CaCDPK15, indicating that CaWRKY40 is not a direct target of CaCDPK15 (Supplementary Fig. S2).

### Effect of transient *CaWRKY40* expression on *CaCDPK15* transcriptional expression

**Figure 6:**
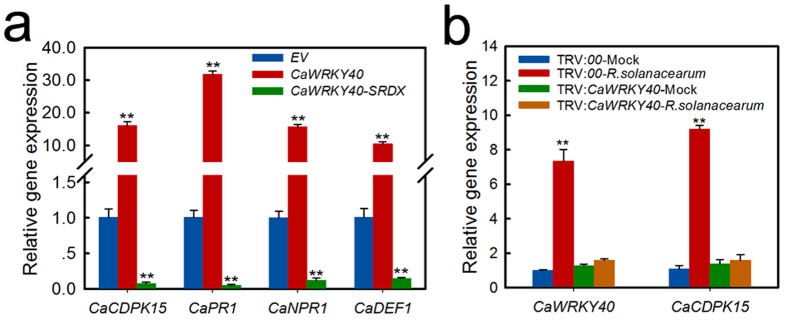
The transcriptional expression of *CaCDPK15* was upregulated by transient overexpression of *CaWRKY40* but downregulated by *CaWRKY40-SRDX*. (**a**) The effect of transient overexpression of *CaWRKY40* and *CaWRKY40-SRDX* on the transcript level of *CaCDPK15* and immunity associated marker genes *CaPR1*, *CaNPR1* and *CaDEF1* in pepper leaves at 24 hpi. (**b**) The transcriptional expression of *CaCDPK15* was downregulated significantly in *CaWRKY40-*silenced pepper plants inoculated by *R. solanacearu*m after 24 hours. Expression values are normalized by the expression levels of *CaACTIN* and *18s rRNA.* (**a,b**) Data are the means ± SD from at least three independent experiments. Asterisks indicate statistically significant differences compared with *EV* ([**a**]) and the treatment of MgCl_2_ (Mock, [**b**]). (*t*-test, **P < 0.01).

More than 7 W-boxes were identified in the *CaCDPK15* promoter, suggesting that CaWRKY40 might act as a regulator of *CaCDPK15*. To test this hypothesis, the transcriptional expression of *CaCDPK15* was determined in pepper leaves transiently overexpressing *CaWRKY40* or its repressor version *CaWRKY40*-*SRDX* by performing real-time RT-PCR analysis. The results showed that transient *CaWRKY40* overexpression significantly activated the expression of *CaCDPK15* and the immunity-associated marker genes *CaNPR1*, *CaPR1*, and *CaDEF1*. By contrast, the expression of *CaCDPK15*, *CaNPR1*, *CaPR1*, and *CaDEF1* was significantly reduced by *CaWRKY40-SRDX* overexpression (Fig. 6a). Consistently, VIGS-mediated *CaWRKY40* silencing in pepper plants significantly downregulated *CaCDPK15* transcriptional expression in response to RSI (Fig. 6b).

### ChIP analysis of CaWRKY40 binding to the *CaCDPK15* promoter

**Figure 7:**
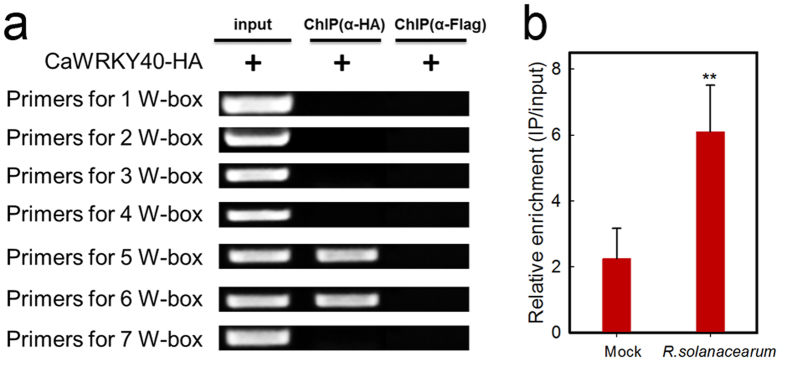
The binding of CaWRKY40 to the promoter of *CaCDPK15* by Chromatin immunoprecipitation (ChIP). (**a**) Binding of CaWRKY40 to the promoter of *CaCDPK15* by ChIP with different pairs of primers according to flanking sequences of different W-boxes. GV3101 cells containing the construct of 35S:*CaWRKY40-HA* was inoculated to the pepper leaves, which are harvested at 48 hpi for preparation of chromatin for ChIP assay, the immunoprecipitated DNA was used as template for PCR with specific primer pairs designed according to the seven W-boxes. Lanes 1, input (total DNA-protein complex); lanes 2, (DNA-protein complex) immunoprecipitated with anti-HA antibody (α-HA), the anti-Flag antibody (α-Flag) was used as a negative control to discriminate the possible unspecific IP in HA-IgG. (**b**) The binding of CaWRKY40 to the promoter of *CaCDPK15* was enhanced by RSI. GV3101 cells containing the construct of 35S:*CaWRKY40-HA* was inoculated to the pepper leaves, 24 hours later, the leaves were further inoculated with *R. solanacearum,* 24 hours later, the leaves were harvested for preparation of chromatin for ChIP assay, and a specific primer pair was used in the real-time RT-PCR. Data are the means ± SD from at least three independent experiments. Asterisks indicate statistically significant differences compared with the treatment of MgCl_2_ (Mock, **[b]**). (*t*-test, **P < 0.01).

As CaWRKY40 significantly activated *CaCDPK15* expression, we speculated that CaWRKY40 might act as a TF in directly modulating *CaCDPK15* expression. We tested this hypothesis by performing ChIP to determine if CaWRKY40 binds to the *CaCDPK15* promoter. For this experiment, GV3101 cells containing the p35S:*CaWRKY40-HA* construct or the empty vector were infiltrated into pepper (GZ03) leaves, which were harvested at 48 hpi for chromatin isolation. The isolated chromatin was randomly sheared into fragments with lengths of 300−500 base pairs, and chromatin fragments that bound to CaWRKY40 were immunoprecipitated using the HA antibody. The resulting DNA fragments were isolated and used as templates for PCR analysis with specific primer pairs. The results showed that only the primer pairs flanking the fifth and sixth W-boxes produced amplified products, suggesting that CaWRKY40 directly binds to the *CaCDPK15* promoter (Fig. 7a). To test the effect of RSI on CaWRKY40 binding to the *CaCDPK15* promoter, ChIP analysis was performed during RSI. The real-time RT-PCR results showed that the infected pepper leaves had higher enrichment of CaWKRY40 in the *CaCDPK15* promoter compared with that of the mock control (Fig. 7b).

## Discussion

Although CDPKs and WRKYs have both been implicated in pathogen attack^45^, the molecular linkage between these two proteins has not been established. We provide strong evidence that CaCDPK15 forms a positive-feedback loop with CaWRKY40 during RSI in pepper, and previously established that CaWRKY40 is a positive regulator of pepper’s response to RSI^36^.

Accumulating evidence indicates that upregulated genes responding to plant defense signaling can have important roles in disease resistance^46^. The *CaCDPK15* promoter contains the following *cis*-elements: 1 SA-responsive TCA element, 1 ethylene-responsive ERE box, and 7W-boxes. These *cis* elements are frequently involved in plant immunity responses^47^. Transgenic tobacco expressing p*CaCDPK15:GUS* consistently exhibited significantly inducible GUS expression in response to RSI, suggesting that CaCDPK15 might act as a positive regulator in pepper’s response to RSI. This possibility was confirmed by loss-of-function experiments, which showed that *CaCDPK15* silencing significantly reduced pepper resistance to RSI, and significantly down-regulated the expression of the immunity marker genes *CaNPR1*, *CaPR1*, and *CaDEF1*. By contrast, transient *CaCDPK15* overexpression in pepper plants triggered HR-mimicking cell death, enhanced electrolyte leakage, and enhanced accumulation of H_2_O_2_. Ca^2+^ is a ubiquitous signal in plant defense responses to biotic and abiotic stresses^48^,^49^, and CDPKs are one of the Ca^2+^ sensors that relay Ca^2+^ signatures to downstream components via protein phosphorylation and transcriptional reprogramming^45^. These combined results suggest that CaCDPK15 acts as a positive regulator of pepper’s response to RSI.

Our previous work showed that *CaWRKY40* was upregulated in response to RSI and high temperature/high humidity, and functioned as a positive regulator in pepper’s response to these two stresses^36^. *CaWRKY40* and *CaCDPK15* have similar expression patterns and functions in pepper’s response to RSI, which suggests a close relationship between these two genes and their encoded proteins. This was corroborated by data showing that the transcriptional expression of *CaWRKY40* was upregulated by transient *CaCDPK15* overexpression in pepper plants, and was significantly down-regulated by VIGS-mediated *CaCDPK15* silencing. Ca^2+^ influx occurs very early during stress challenge. CDPK proteins localized in the plasma membrane and/or cytoplasm are expected to be involved in early signaling pathways that respond to biotic and abiotic stresses^49^. CaCDPK15 is believed to act as an upstream regulator of *CaWRKY40*. Similar molecular linkages between CDPKs and WRKYs have been reported previously^50^,^51^. For example, the overexpression of wheat (*Triticum aestivum*) *TaCPK2-A* in rice (*Oryza sativa*) promoted *OsWRKY45-1* expression, which is a TF involved in resistance to fungi and bacteria, by regulating genes involved in JA and SA signaling^50^. In *Arabidopsis*, the CDPK4/5/6/11 isoforms phosphorylate a specific subgroup of WRKY TFs (WRKY8/28/48) that regulate crucial transcriptional reprogramming that restricts pathogen growth^51^. However, unlike these CDPK and WRKY proteins, this study provided evidence that CaCDPK15 and CaWRKY40 do not directly interact with each other, and CaCDPK15 does not phosphorylate CaWRKY40; co-IP detected no interaction between CaCDPK15 and CaWRKY40, and CaCDPK15 localized to nuclei. Therefore, CaCDPK15 might phosphorylate and activate other TFs that target CaWRKY40. Further identification of possible CaCDPK15 interactors that subsequently target CaWRKY40 might provide new insights into the mechanism of pepper immunity mediated by CaCDPK15 and CaWRKY40.

We showed that CaCDPK15 modulates *CaWRKY40* expression. Unexpectedly, we found that *CaCDPK15* expression in pepper plants was transcriptionally upregulated by transient *CaWRKY40* overexpression, whereas it was downregulated by transient overexpression of *CaWRKY40-SRDX* (repressor) and by *CaWRKY40* silencing. This suggests that there is a positive-feedback loop between CaCDPK15 and CaWRKY40. The presence of 7W-boxes in the *CaCDPK15* promoter indicated that WRKY TFs might directly transcriptionally regulate *CaCDPK15* expression. Our ChIP analysis data revealed that CaWRKY40 binds to the *CaCDPK15* promoter, which was significantly enhanced by RSI. These results strongly suggest that CaWRKY40 acts as a direct TF in the transcriptional modulation of *CaCDPK15* expression. Similar positive-feedback loops in plant responses to stresses including pathogen attack have been reported^52^,^53^,^54^. For example, SA was reported to act in a positive-feedback loop with ACCELERATED CELL DEATH6 (ACD6) to potentiate plant responsiveness to pathogen-associated molecular patterns (PAMPs)^52^; HEAT SHOCK PROTEIN101 and HEAT STRESS-ASSOCIATED 32-KD PROTEIN form a positive-feedback loop that modulates long-term acquired thermotolerance^53^; and positive-feedback regulation by ABA on LOS6/ABA1 expression provides a quick adaptation strategy for plants under osmotic stress^54^. In general, plant defense systems tend to focus on early stress-mediated events, and these positive-feedback loops may be important for amplifying defense signaling^55^. Our data also showed that exogenous application of SA, MeJA, ABA, and ETH synergistically upregulated *CaCDPK15* expression, which is consistent with their effects on *CaWRKY40* expression^36^. These results strongly suggest a molecular linkage between CaCDPK15 and CaWRKY40.

In our previous study, CaWRKY40 positively regulated pepper response to RSI and plant thermotolerance under high humidity, which is important for plant adaption to conditions that promote the invasion and growth of soil-borne pathogens. Although the present study focused on the role of *CaCDPK15* in pepper immunity, there were indications that *CaCDPK15* might be involved in thermotolerance under high humidity. For example, we identified an HSE element in the *CaCDPK15* promoter, determined that p*CaCDPK15*-driven GUS expression also was activated by heat stress treatment. and found that *CaCDPK15* silencing impaired plant thermotolerance and downregulated the expression of the thermotolerance-associated marker gene *CaHSP24*. *CaHSP24* expression was consistently upregulated by *CaCDPK15* expression (data not shown).

Collectively, our data indicate that *CaCDPK15* expression is upregulated by RSI, which indirectly activates the transcriptional expression of downstream *CaWRKY40*. Likewise, transcriptional expression of *CaWRKY40* directly activates the transcriptional expression of *CaCDPK15*. This generates a positive-feedback loop that would rapidly amplify plant signaling in response to RSI and efficiently activate plant defense responses.

## Methods

### Plant materials and growth conditions

Seeds of the pepper (*Capsicum annuum*) cultivar GZ03 and *Nicotiana benthamiana* were provided by the pepper breeding group at Fujian Agriculture and Forestry University. The seeds were sown in a soil mix [peat moss:perlite, 2:1 (v/v)] in plastic pots, and were placed in a greenhouse at 25 °C, 60–70 μmol photons m^−2^ s^−1^, 70% relative humidity, and a 16-h light/8-h dark photoperiod.

### Pathogens and inoculation procedures

*R. solanacearum* strain FJC100301 was isolated previously in our lab and amplified according to the method of Dang *et al.*^36^. The bacterial cell culture was diluted to 10^8^ cfu ml^−1^ (OD_600_ = 0.8) with 10 mM MgCl_2_. Pepper plants were inoculated by infiltrating 10 ml of the resulting *R. solanacearum* suspension into the third leaves from the apical meristem using a syringe with a needle. The leaves were harvested at the indicated time points for the analysis of GUS activity, ChIP, or RNA.

### Treatment of plants with exogenous phytohormones and RSI

Pepper plants at the four-leaf stage were sprayed with 1 mM salicylic acid, 100 μM methyl jasmonate, 100 μM abscisic acid, or 100 μM ethephon. Mock-treated plants were sprayed with a corresponding solvent or sterile ddH_2_O. To study the relative *CaCDPK15* transcript levels in response to *R. solanacearum* infection, pepper plants were inoculated at the eight-leaf stage by injecting 10 ml of bacterial suspension (10^8^ cfu ml^−1^) or distilled sterile 10 mM MgCl_2_, and then harvested at different time points.

### Analysis of *CaCDPK15* subcellular localization

The full-length cDNA of *CaCDPK15* was cloned into the plant expression vector pMDC83 downstream of the two CaMV35S promoters and in-frame with GFP using the Gateway cloning system (Invitrogen). The 35S:*CaCDPK15-GFP* and 35S:*GFP* (used as a control) constructs were transformed into *Agrobacterium tumefaciens* strain GV3101, which was cultured in induction medium (10 mM ethanesulfonic acid, pH 5.4, 10 mM MgCl_2_, and 200 mM acetosyringone), harvested when the OD_600_ was approximately 1.0, and diluted to OD_600_ = 0.8. Bacterial suspensions expressing p35S:*CaCDPK15-GFP* and p35S:*GFP* were injected into *Nicotiana benthamiana* leaves using a syringe without a needle. GFP fluorescence was imaged using laser confocal fluorescence microscopy (Leica TCS SP8) with an excitation wavelength of 488 nm and a 505–530 nm band-pass emission filter.

### Histochemical staining

Staining with trypan blue and DAB was performed according to the previously published method of Choi *et al.*^56^. For trypan blue staining, pepper leaves were boiled in trypan blue staining solution for 2 min, left at room temperature for 8 h, transferred into a chloral hydrate solution (2.5 g of chloral hydrate dissolved in 1 mL of distilled water), and boiled for 20 min to destain. After multiple changes of chloral hydrate solution to reduce the background, samples were mounted in 70% glycerol. For DAB staining, the leaves were stained overnight in 1 mg ml^−1^ DAB. The stained leaves were cleared by boiling in lactic acid:glycerol:absolute ethanol [1:1:3 (v/v/v)], and then destained overnight in absolute ethanol. Representative images of DAB and trypan blue staining were photographed with a light microscope (Leica, Wetzlar, Germany).

### Virus-induced gene silencing (VIGS) of *CaCDPK15* in pepper plants

The tobacco rattle virus (TRV)-based VIGS system was employed to silence *CaCDPK15*. The PYL192 and PYL279 VIGS vectors were described previously^57^. A fragment of the transcribed region of *CaCDPK15* was amplified using gene-specific primers (5′-GGGGACAAGTTTGTACAAAAAAGCAGGCTTCTTTTCTTTTCGC CCTTTA-3′ and 5′-GGGGACCACTTTGTACAAGAAAGCTGGGTCAATGAACT CCATCCAGCA-3′), and cloned into the PYL279 VIGS vector using the Gateway cloning system (Invitrogen). The PYL192 and PYL279 vectors were with or without *CaCDPK15*, respectively. PYL279 contained a 250- or 500-bp *PDS* fragment. The PYL192 and PYL279 vectors were transformed into *A. tumefaciens* strain GV3101. *Agrobacterium* harboring PYL192 with PYL279 (PYL192 vector with PYL279 as TRV:*00*), PYL279-*CaCDPK15* (PYL192 with PYL279-*CaCDPK15* as TRV:*CaCDPK15*), or PYL279-*PDS* (OD_600_ = 1.0) were mixed at a 1:1 ratio, and the mixture was infiltrated into cotyledons of 2-week-old pepper plants using a 1 ml sterile syringe without a needle. The *Agrobacterium*-inoculated pepper plants were grown for 2–3 weeks in a growth chamber at 16 °C (in darkness for the first 56 h) with 45% relative humidity, and then transferred into a growth room at 25 ± 2 °C, 60–70 μmol photons m^−2^ s^−1^, 70% relative humidity, and a 16-h light/8-h dark photoperiod.

### Transient expression assay of *CaCDPK15*

*Agrobacterium tumefaciens* strain GV3101 harboring the pK7WG2*-CaCDPK15* vector was cultured to OD_600_ = 1.0 in induction medium (10 mM ethanesulfonic acid, pH 5.7, 10 mM MgCl_2_, and 200 mM acetosyringone) and diluted to OD_600_ = 0.8. The diluted culture was injected into pepper or *Nicotiana benthamiana* leaves using a syringe without a needle. The plants were kept in a growth room for 2 days, and then the injected leaves were harvested for further use.

### Co-immunoprecipitation assay

To construct vectors for co-IP analysis, *CaCDPK15* or *CaWRKY40* in the pDONR vector was directly introduced into the destination vectors p35S:*HA* (pEarleyGate 201) and p35S:*Flag* (pEarleyGate 202) to generate p35S:*CaCDPK15-HA* and p35S:*CaWRKY40-Flag* by the LR reaction (Invitrogen). Plasmids were transformed into *Agrobacterium* strain GV3101, and cells harboring p35S:*CaCDPK15-HA* and p35S:*CaWRKY40-Flag* were simultaneously infiltrated into leaves of *N. benthamiana* plants. Leaves were harvested at 48 h after infiltration (hai), and total proteins were extracted using protein extraction buffer [10% glycerol, 25 mM Tris-HCl, pH 7.5, 150 mM NaCl, 1 mM EDTA, 2% Triton X-100, 10 mM DTT, 1× complete protease inhibitor cocktail (Sigma-Aldrich, St. Louis, MO, USA), and 2% (w/v) polyvinylpolypyrrolidone]^58^. Extracted proteins were incubated with monoclonal anti-HA magnetic beads at 4 °C overnight. Beads were then collected with a magnet and washed three times with protein extraction buffer. Eluted proteins were immunoblotted using anti-HA-peroxidase antibody.

### Quantitative real-time PCR

To determine the relative transcript levels of selected genes, real-time PCR was performed with specific primers (Supplementary Table S1 and Table S2) according to the manufacturer’s instructions for the BIO-RAD Real-time PCR system (Foster City, CA, USA) and the SYBR Premix Ex Taq II system (TaKaRa Perfect Real Time). Total RNA was isolated from pepper plants using TRIzol reagent (Invitrogen), and was reverse-transcribed using the PrimeScript RT-PCR kit (TaKaRa, Dalian, China)^59^. A 10-fold dilution of the resulting cDNA was amplified using the SYBR Premix Ex Taq II kit and the BIO-RAD Real-time PCR system in a 10 μl volume with the following program: 95 °C for 30 s; 40 cycles of 95 °C for 5 s and 60 °C for 34 s; and 95 °C for 15 s. Amplification of the target genes was monitored every cycle by SYBR green fluorescence. The Ct (threshold cycle) value, which is defined as the real-time PCR cycle at which a statistically significant increase of reporter fluorescence was first detected, was used as a measure for the starting target gene copy number. Three replicates of each experiment were performed. Data were analyzed by the Livak method and expressed as a normalized relative expression level (2^−ΔΔCT^) of the respective genes. The relative transcript levels were normalized with respect to the transcript levels of *CaActin* and *18* *s rRNA*. In each case, three technical replicates were performed for at least three independent biological replicates.

### Chromatin immunoprecipitation analysis

The 35S:*CaWRKY40-HA* and 35S:*CaCDPK15-Flag* constructs were generated by Gateway cloning (Invitrogen)^60^, and were transformed into *Agrobacterium* strain GV3101. GV3101 cells containing 35S:*CaWRKY40-HA* and 35S:*CaCDPK15-Flag* were co-infiltrated at a ratio of 1:1 or infiltrated individually into pepper leaves, which were harvested at 48 hpi for chromatin preparation. ChIP was performed according to standard protocols. Briefly, approximately 2 g of pepper leaves was treated with either 10 mM bithionol sulfoxide or DMSO (solvent control) for 16 h and subsequently fixed with 1.0% formaldehyde for 5 min. Antibody against HA or FLAG (Santa Cruz Biotechnology) were used for immunoprecipitation. Protein-A-agarose beads were blocked with salmon sperm DNA and used to pull down the protein-DNA complex. Equal amounts of starting plant material and the ChIP products were used for PCR or real-time PCR with specific primers for the promoters of *CaCDPK15*, *CaPR1*, *CaNPR1*, and *CaDEF1* (Supplementary Table S3).

## Additional Information

**How to cite this article**: Shen, L. *et al.* CaCDPK15 positively regulates pepper responses to *Ralstonia solanacearum* inoculation and forms a positive-feedback loop with CaWRKY40 to amplify defense signaling. *Sci. Rep.* **6**, 22439; doi: 10.1038/srep22439 (2016).

## Supplementary Material

Supplementary Information
