# Supplementary material for: CaCDPK15 positively regulates pepper responses to Ralstonia solanacearum inoculation and forms a positive-feedback loop with CaWRKY40 to amplify defense signaling
Source: Sci Rep. 2016 Mar 1;6:22439. doi: 10.1038/srep22439 (PMC4772545; doi:10.1038/srep22439)
Supplement: Supplementary Information [file srep22439-s1.doc]

**CaCDPK15 positively regulates pepper responses to *Ralstonia* *solanacearum* inoculation and forms a positive-feedback loop with CaWRKY40 to amplify defense signaling**

Lei Shen^1,2,+^, Sheng Yang^1,2,+^, Tong Yang^1,3^, Jiaqi Liang^1,3^, Wei Cheng^1,2^, Jiayu Wen^1,2^, Yanyan Liu^1,2^, Jiazhi Li^1,3^, Lanping Shi^1,2^, Qian Tang^1,2^, Wei Shi^1,2^, Jiong Hu^1,2^, Cailing Liu^1,2^, Yangwen Zhang^1,2^, Shaoliang Mou^1,3^, Zhiqin Liu^1,2^, , Hanyang Cai^1,3^, Li He^4^, Deyi Guan^1,2^, Yang Wu^4^,*, Shuilin He^1,2^,*

**SupplementaryData**

**Supplementary** **Figures**

**
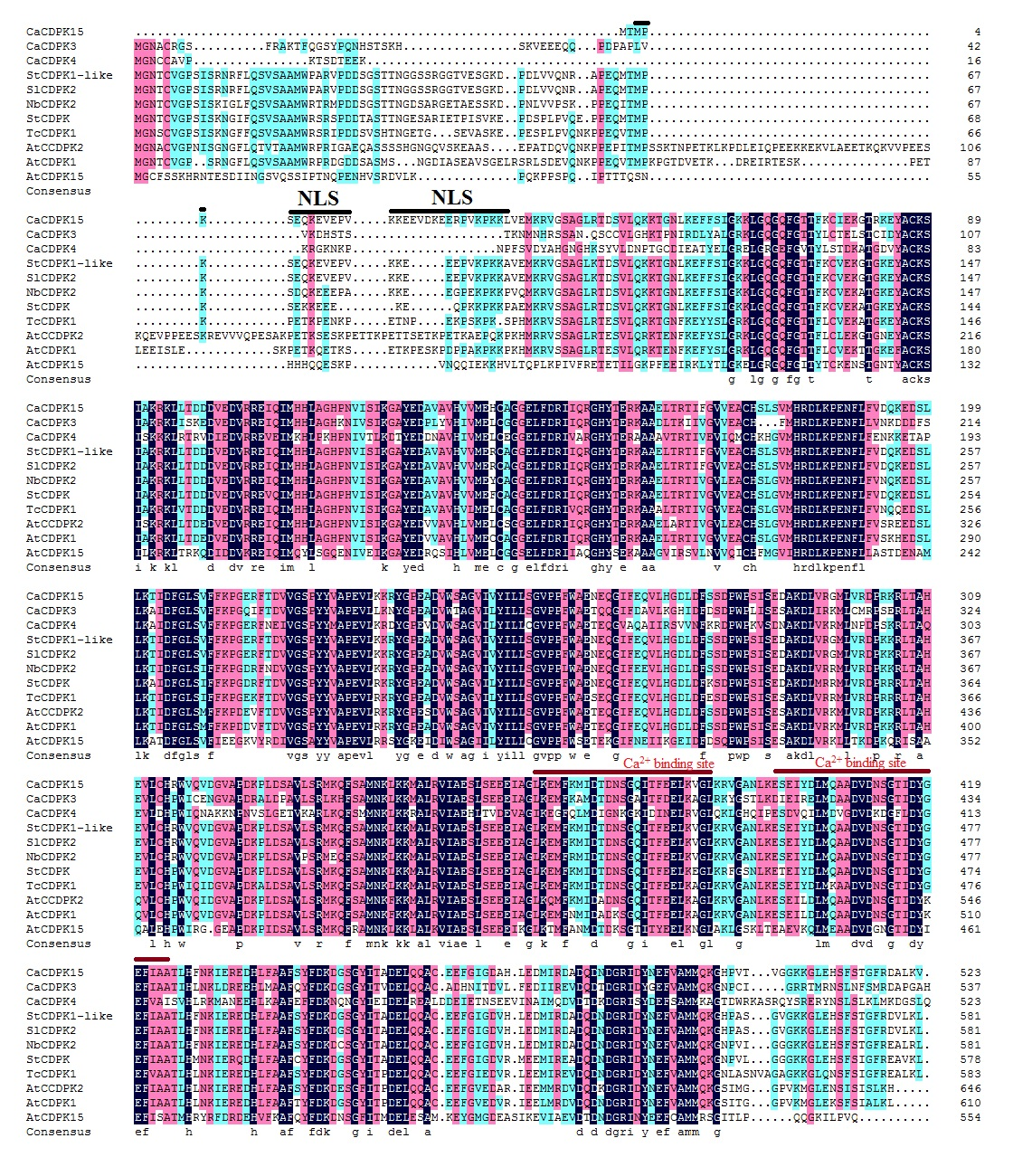
**

**Fig. S1.** Comparison of amino acid sequences of CabZIP63 with representative related proteins. Including *StCDPK1*-like (*Solanum tuberosum*, NP_001275230), *SlCDPK* (*Solanum lycopersicum*, NP_001234784), *NbCDPK2* (*Nicotiana benthamiana*, [CAC83000](http://www.ncbi.nlm.nih.gov/protein/CAC83000)), *StCDPK* (*Solanum tuberosum*, [BAB63463](http://www.ncbi.nlm.nih.gov/protein/BAB63463)), *TcCDPK1* (*Theobroma cacao*, [XP_007010504](http://www.ncbi.nlm.nih.gov/protein/XP_007010504)), *AtCCDPK2* (*Arabidopsis thaliana*, NP_187677), *AtCDPK15* (*Arabidopsis thaliana*, [NP_193925](http://www.ncbi.nlm.nih.gov/protein/NP_193925)), *CaCDPK3* (*Capsicum annuum*, AY295081), *CaCDPK4* (*Capsicum annuum*, AY904339) and *AtCDPK1* (*Arabidopsis thaliana*, D21805). The amino acid sequences which were marked by the black lines and blue lines represented nuclear locational signal (NLS) and Ca^2+^ binding site, respectively. Green shading, 50%-75% identity; red shading, 75%-100% identity; black shading, 100% identity. The alignment was carried out using DNAMAN5.

**
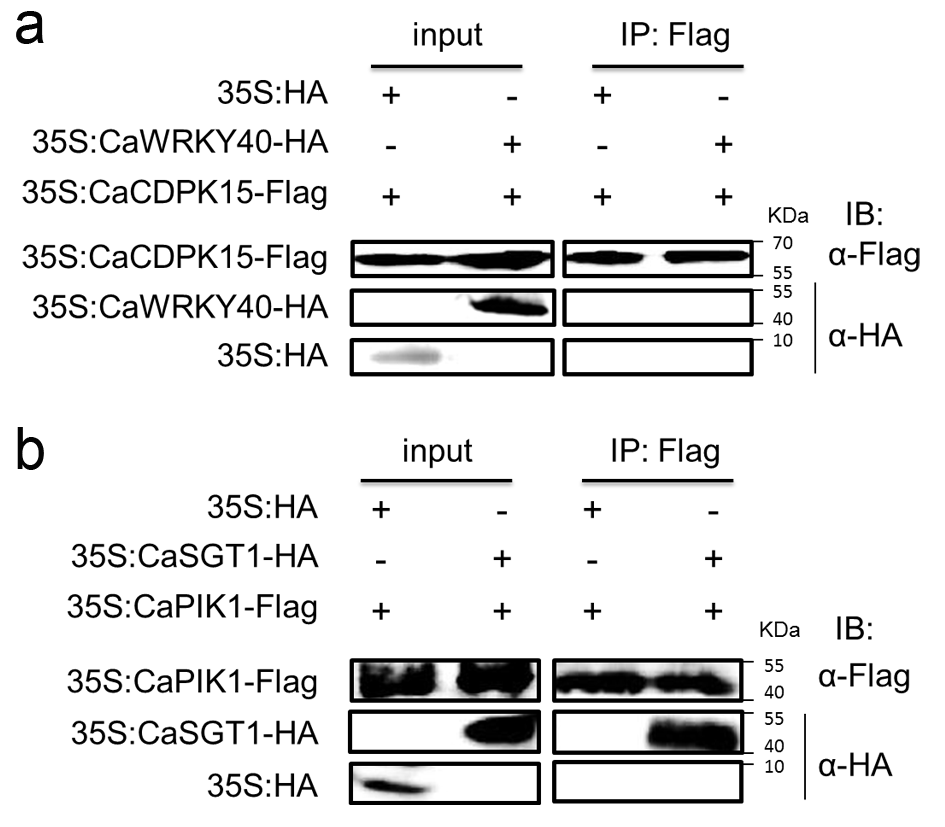
**

**Fig. S2.** Assay of the possible interaction between CaCDPK15 and CaWRKY40 in N. benthamiana leaves. **(a)** Co-IP and immunoblotting (IB) of 35S:HA or 35S:CaWRKY40-HA and 35S:CaCDPK15-Flag proteins coexpressed in N. benthamiana leaves. 35S:HA was used as a negative control. **(b)** Interaction between 35S:CaSGT1-HA and 35S:CaPIK1-Flag by co-IP and immunoblotting (IB) in the same system to **(a)** was used as a positive control.

**Supplementary Tables**

**Table S1.** Pepper primers for generation of various gene constructs used in this study.

| **Gene** | **Accession no.** | **Forward primers (5’ to 3’)** | **Reveres primers (5’ to 3’)** | **Size (bps)** |
| --- | --- | --- | --- | --- |
| *CaCDPK15* ^1^ | CA05g03200 | GGGGACAAGTTTGTACAAAAAAGCAGGCTTCATGACAATGCCTAAGTCGGAA | GGGGACCACTTTGTACAAGAAAGCTGGGTCTTAGACCTTTAGAGCGTCTCT | 1572 |
| *CaCDPK15* ^2^ | CA05g03200 | GGGGACAAGTTTGTACAAAAAAGCAGGCTTCATGACAATGCCTAAGTCGGAA | GGGGACCACTTTGTACAAGAAAGCTGGGTCGACCTTTAGAGCGTCTCTGA | 1569 |
| *CaCDPK15 ^3^* | CA05g03200 | GGGGACAAGTTTGTACAAAAAAGCAGGCTTCTTTTGAACAGAGACAAGTGTG | GGGGACCACTTTGTACAAGAAAGCTGGGTCCTGTTCTGGTGCCCTATTTTG | 2000 |
| *CaCDPK15* ^4^ | CA05g03200 | GGGGACAAGTTTGTACAAAAAAGCAGGCTTCTTTTCTTTTCGCCCTTTA | GGGGACCACTTTGTACAAGAAAGCTGGGTCAATGAACTCCATCCAGCA | 179 |

^1^ Primers for full-length *CaCDPK15* cloning.

^2^ Primers for the vector constructing of 35S:*CaCDPK15-83*.

^3^Primers for full-length *CaCDPK15* promoter cloning.

^4^Primers for the vector constructing of TRV:*CaCDPK15*.

**Table S2.** Pepper gene-special primers for real-time RT-PCR used in this study.

| **Gene** | **Accession no.** | **Forward primers (5’ to 3’)** | **Reveres primers (5’ to 3’)** |
| --- | --- | --- | --- |
| *CaCDPK15* | CA05g03200 | TTTTCTTTTCGCCCTTTA | AATGAACTCCATCCAGCA |
| *CaWRKY40* | AAX20040.1 | AACTTGGATGTTGTGCCTGGA | CTGTAACCTTGGCTTTTATGTGC |
| *CaPR1* | AF348141.1 | GCCGTGAAGATGTGGGTCAATGA | TGAGTTACGCCAGACTACCTGAGTA |
| *CaNPR1* | X61679.1 | ACTTCTTCGCCGACGCCAAG | GCCAACACATTCACCAGAGCATC |
| *CaDEF1* | AF442388 | GTGAGGAAGAAGTTTGAAAGAAAGTAC | TGCACAGCACTATCATTGCATACAATTC |
| *CaPO2* | DQ489711 | ATGGCAGAGAAAACCACCAGCA | TCAAAAAAAAGTGACCTCCTTTCTGT |
| *CaHIR1* | AY529867 | GACAAAGCTAATGAAGCATTCTAC | GGTGTCGAAGTACTGGGTTACC |
| *CaACTIN* | GQ339766 | AGGGATGGGTCAAAAGGATGC | GAGACAACACCGCCTGAATAGC |
| *18s rRNA* | EF564281 | CCGGTCCGCCTATGGTGTGCACCGGTCGTC | GCAGTTGTTCGTCTTTCATAAATCCAAGAA |

**Table S3.** Pepper gene-special primers for ChIP-PCR or real-time RT-PCR used in this study.

| **Primer name** | **Forward primers (5’ to 3’)** | **Reveres primers (5’ to 3’)** |
| --- | --- | --- |
| pCaCDPK151 W-box | ATCTTGATTTGGTTAAACTTG | AAATCAAGAATCAAGCTACA |
| pCaCDPK152 W-box | ACTTTTGAGTTTGGTGAAAA | ACACACACACAAAAAAAAAA |
| pCaCDPK153 W-box | TTTTTTGAGTATAAGATTGT | CCTTTTTCACCAAACTCAAA |
| pCaCDPK154 W-box | TTGAACAAGTTCTTGATTTT | AGTAAAATCAACAACTTGTT |
| pCaCDPK155 W-box | ATCTTGATTTGGTTAAACTTG | AAATCAAGAATCAAGCTACA |
| pCaCDPK156 W-box | TTTTCCTTTGTAACCTTAGTT | AAGACAAAGTAAAAGCCAAA |
| pCaCDPK157 W-box | CTATTGTGAGTGATATTTG | ATTATAGAAAAAAATCTACT |
| pCaPR1 1W-box | ATATAAATCCTTATTATAAG | TAGCAATACCTGGTGAACCA |
| pCaNPR1 1W-box | CTAACCCATACATTATTATT | GGGGGCAGAGAAGAAGGGAA |
| pCaDEF1 2W-box | GGGCATAGCCGTGACCAACG | TCGAGTCGCGGCTCCACGCG |
